# Targeting polarized phenotype of microglia via IL6/JAK2/STAT3 signaling to reduce NSCLC brain metastasis

**DOI:** 10.1038/s41392-022-00872-9

**Published:** 2022-02-23

**Authors:** Yu Jin, Yalin Kang, Minghuan Wang, Bili Wu, Beibei Su, Han Yin, Yang Tang, Qianxia Li, Wenjie Wei, Qi Mei, Guangyuan Hu, Veronika Lukacs-Kornek, Jian Li, Kongming Wu, Xianglin Yuan, Wei Wang

**Affiliations:** 1 Department of Oncology, Tongji Hospital, Tongji Medical College, Huazhong University of Science and Technology, Wuhan, 430030 Hubei Province China; 2 Department of Neurology, Tongji Hospital, Tongji Medical College, Huazhong University of Science and Technology, Wuhan, 430030 Hubei Province China; 3 Institute of Experimental Immunology, University Clinic of Rheinische Friedrich-Wilhelms-University, Bonn, Germany

**Keywords:** Lung cancer, Metastasis, CNS cancer, Molecular medicine

## Abstract

Tumor-associated macrophages have emerged as crucial factors for metastases. Microglia are indispensable components of the brain microenvironment and play vital roles in brain metastasis (BM). However, the underlying mechanism of how activated microglia promote brain metastasis of non-small cell lung cancer (NSCLC) remains elusive. Here, we purified cell lines with brain-metastatic tropism and employed a co-culture system to reveal their communication with microglia. By single-cell RNA-sequencing and transcriptome difference analysis, we identified IL6 as the key regulator in brain-metastatic cells (A549-F3) to induce anti-inflammatory microglia via JAK2/STAT3 signaling, which in turn promoted the colonization process in metastatic A549-F3 cells. In our clinical samples, patients with higher levels of IL6 in serum showed higher propensity for brain metastasis. Additionally, the TCGA (The Cancer Genome Atlas) data revealed that NSCLC patients with a lower level of IL6 had a longer overall survival time compared to those with a higher level of IL6. Overall, our data indicate that the targeting of IL6/JAK2/STAT3 signaling in activated microglia may be a promising new approach for inhibiting brain metastasis in NSCLC patients.

## Introduction

To date, lung cancer has the highest mortality rate among all malignancies worldwide.^1^ There are different types of lung cancer, and non-small cell lung cancer (NSCLC) accounts for 85% of all lung cancers cases.^2^ Lung adenocarcinoma, a subtype of NSCLC, is the most common lung cancer subtype. Metastasis is a major determinant of patient prognosis, with the most common metastatic site in the brain. It is estimated that 30–50% of NSCLC patients have the brain metastasis (BM) during disease progression.^3^ Patients with NSCLC-BM have a very poor prognosis due to the limited efficacy of therapeutic options, such that the total median survival time of NSCLC-BM patients is in the range of 1.5 to 9.5 months.^4,5^ Unfortunately, molecular mechanisms underlying BM remain elusive, particularly with respect to factors promoting NSCLC cell survival in the circulation and colonization in the brain. Therefore, investigation of the mechanisms of metastatic colonization is imperative for the identification of new therapeutic targets and subsequent improvement of the life quality for NSCLC-BM patients.

With regard to the mechanism of BM, studies have focused on the initial stage of metastasis. That is, how cancer cells leave the original site to form “seeds” with cancer stem cell characteristics and how they penetrate the blood-brain barrier (BBB).^6,7^ The most extensively researched process in this context is the epithelial-to-mesenchymal transition (EMT).^8^ During EMT, epithelial cells transform into mesenchymal stem cells, and their ability to metastasize considerably increases, allowing tumor cells to colonize a distant metastatic site.^9,10^ Once tumor cells reach a distant organ, reversal of the EMT occurs, known as the mesenchymal-to-epithelial transition (MET).^10,11^ Previous research has demonstrated MET in breast and colorectal cancer metastases,^12,13^ but the process of colonization of NSCLC in the brain remains unclear. Herein, the current work investigated NSCLC metastatic cell colonization of the brain, focusing on the MET.

Interaction of cancer cells with the tumor microenvironment is essential for metastatic colonization.^14,15^ Microglia, as the resident macrophages of the brain, are indispensable components of the brain microenvironment to participate in processes of innate immunity and maintain central nervous system homeostasis.^16,17^ The role of microglia in primary brain tumors, such as glioblastoma and pilocytic astrocytoma,^18,19^ has been widely investigated. However, their involvement in BM, which are more common in clinical practice, is poorly understood. Research has revealed an abundance of activated microglia infiltrating metastatic lesions of lung cancer patients, but the research on the mechanism is limited.^20^ As tissue-specific macrophages, microglia exhibit common macrophage characteristics. It is widely accepted that macrophages have two distinct activation states, namely the classical pro-inflammatory M1 state (with markers iNOS and CD86) and the anti-inflammatory M2 state (with markers CD206 and Arg1).^21,22^ M2 tumor-associated macrophages (TAMs) have been proven to play a crucial role in tumor progression and metastasis.^23,24^ Moreover, Watabe et al. demonstrated that M2 microglia suppress local immunity and promote breast cancer cell colonization in the brain.^25^ These findings suggest that microglia may be potential therapeutic targets for NSCLC-BM.

Given that microglia exhibit functional plasticity under the stimulation of various microenvironmental factors,^26^ we investigated the communication between tumor cells and microglia during colonization of the brain by metastatic NSCLC cells. In this study, we purified cells selectively metastatic to the brain using a mouse model and established an in vitro co-culture system to study the metastatic process. Furthermore, we confirmed the existence of anti-inflammatory microglia and identified different cell types within BM by single-cell RNA-sequencing. Our study aimed to elucidate the underlying mechanism involving activated microglia and identify therapeutic targets for preventive treatment in NSCLC-BM.

## Results

### Alteration of the microglia phenotype in the microenvironment of BM

**Fig. 1:**
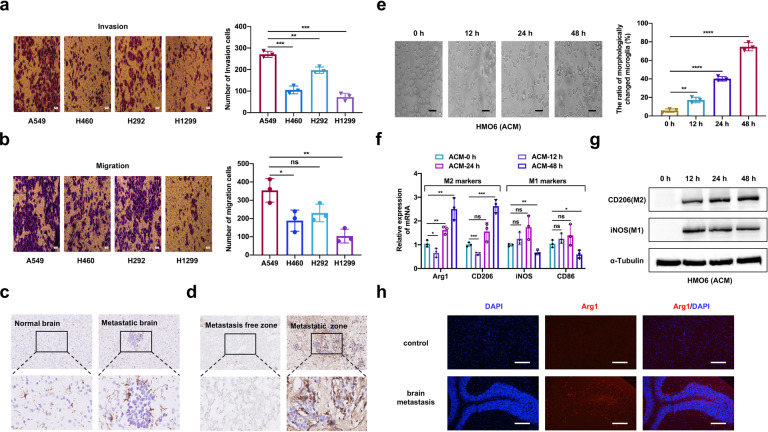
Human NSCLC A549 cells alter phenotype of microglia. **a** Evaluating the ability of invasion in different cell lines by Transwell invasion assay (magnification, 400×). **b** Evaluating the ability of migration in different cell lines by Transwell migration assay (magnification, 400×). **c** Representative images of IHC staining with IBA-1 (brown) and nuclear counterstaining with hematoxylin (blue) were depicted. Left image was from a control mouse and the right image was brain specimens of a mouse with brain metastasis. Top panels: magnification was 200×. Bottom panels were with higher magnification (800×). **d** Human specimens of NSCLC-BM showing metastasis free and positive areas, metastatic zone was surrounded with activated IBA-1^+^ microglia cells. Top panels: magnification was 200×. Bottom panels were with higher magnification (800×). **e** Morphologic changes of HMO6 cells treated with ACM for 0 h, 12 h, 24 h, 48 h were evaluated by phase-contrast microscopy (magnification: 400×). **f** The relative expression of M1-markers (iNOS and CD86) and M2-markers (Arg1 and CD206) mRNA in HMO6 cells treated with ACM for 0 h, 12 h, 24 h, 48 h are detected by quantitative RT-PCR. β-Actin was used to normalize gene expression. **g** Western blot and the corresponding gray value analysis were used to investigate the expression iNOS and CD206 in HMO6 cells cultured in ACM for 0, 12, 24, 48 h. **h** Representative images of immunofluorescence staining with DAPI (blue) for nuclei and Arg1(red) for M2-marker were depicted (magnification: 20×). ACM: A549 cells-conditioned media. Data are mean ± SD. *P* > 0.05 no significant difference; *P < 0.05; **P < 0.01; ***P < 0.001; ****P < 0.0001

To explore the role of microglia in NSCLC brain metastasis, we first assessed the metastatic ability of several commonly used NSCLC cell lines with wild-type EGFR (A549, H460, H292, and H1299) (Fig. 1a, b). Among them, A549 cells exhibited the highest invasion and migratory capacity and were therefore chosen for further investigation. We intracardially inoculated A549 cells into nude mice to establish brain-metastatic model. Brain tissues of mice in the metastatic group and a non-inoculation control group were stained with Iba-1 to analyze the distribution of microglia. Compared to the control group, the treatment group showed abundant microglia with altered morphology and enhanced arborization in brain-metastatic lesions (Fig. 1c). Consistently, Iba-1 immunohistochemistry staining of human NSCLC-BM specimens revealed that activated microglia infiltrated the close vicinity of BM lesion (Fig. 1d).

To investigate the NSCLC cell-microglia interaction, we conducted in vitro cellular experiments to stimulate microglia with the conditioned medium (CM) of A549 cells (ACM) to examine its effect on microglial morphology at different time points. We observed prominent morphological changes, with ramified morphologies turning into bushy shapes after 24 h and 48 h (Fig. 1e). To elucidate the effect of ACM on specific microglial activation, the expressions of pro-inflammatory M1 markers (iNOS and CD86) and anti-inflammatory M2 markers (CD206 and ARG1) were analyzed by qRT-PCR. The expressions of iNOS and CD86 were upregulated at 12 h (*P* < 0.05), while the expression levels of CD206 and Arg1 were elevated at 24 h and 48 h (*P* < 0.05) (Fig. 1f). Western blot analysis showed that iNOS and CD206 levels in HMO6 cells were almost undetectable at 0 h, indicating the quiescent state of microglia (Fig. 1g). Thereafter, the protein level of iNOS was upregulated at 12 h, whereas the protein level of CD206 increased at 24 h and 48 h. The Immunofluorescence analysis show that CD206^+^ microglia were significantly higher in groups treated with ACM for 24 h and 48 h (*P* < 0.05) (Supplementary Fig. S1a, b). All results were consistently reflected in the specimens of NSCLC-BM patients that the CD206^+^ microglia were significantly more than the iNOS^+^ microglia (Fig. 1h and Supplementary Fig. S1c).

### CM of anti-inflammatory microglia promotes colonization by NSCLC cells

**Fig. 2:**
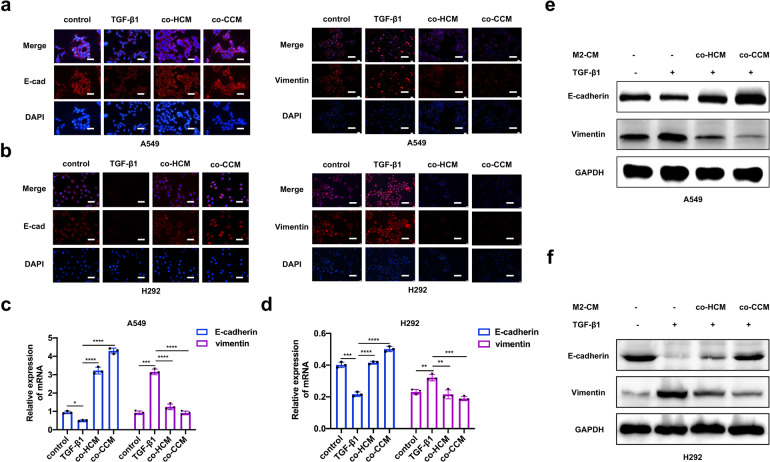
M2-Microglia CM promotes NSCLC cells colonization. **a**, **b** E-cadherin (magnification: 400×) and vimentin (magnification: 200×) expression of A549 and H292 cells were presented by immunofluorescence staining. The blue signal represents the DAPI-stained nuclei. **c** qRT-PCR analysis of E-cadherin and vimentin for A549 cells cultured with different treatment. **d** qRT-PCR analysis of E-cadherin and vimentin for H292 cells cultured with different treatment. **e** Western blot for E-cadherin and vimentin of A549 cells cultured under different conditions. The right panels were gray value analysis. **f** Western blot for E-cadherin and vimentin of H292 cells cultured under different conditions. The right panels were gray value analysis. control: normal A549 cells; TGF-β1: A549 cells treated with TGF-β1 (2.5 ng/ml) for 24 h; co-HCM and co-CCM: A549 cells pretreated with TGF-β1 (2.5 ng/ml) and incubated with the co-cultured CM for 24 h. co-HCM/co-CCM: CM from A549 and HMO6/CHME5 cells co-cultured for 48 h. Data are mean ± SD. *P < 0.05, **P < 0.01, ***P < 0.001, ****P < 0.0001

Given the results described above, we hypothesized that anti-inflammatory microglia promoted colonization by mesenchymal NSCLC cells through the MET. For this, we sought to explore whether alternatively activated microglia could reverse the EMT occurring within the primary tumor. Firstly, we triggered the EMT in A549 and H292 cells via TGF-β1 treatment, which was previously reported to induce the EMT in epithelial tumor cells.^27^ Next, we explored the impact of co-culture CM (co-HCM and co-CCM) in mesenchymal A549 and H292 cells by immunofluorescence analysis. The TGF-β1 reduced E-cadherin expression at prominent cell junctions and upregulated the expression of vimentin, while the co-culture CM (co-HCM and co-CCM) could reverse this process (Fig. 2a, b). To further validate these observations, we conducted qRT-PCR and western blot analyses. Results showed that co-culture CM could induce the MET of mesenchymal-like A549 cells (Fig. 2c, e) and H292 cells (Fig. 2d, f) along with E-cadherin upregulation and vimentin downregulation. These results indicate that anti-inflammatory microglia are sufficient enough to reprogram the cancer cells to undergo MET to acquire the epithelial phenotype and promote metastatic colonization in the brain.

### Single-cell RNA-seq revealed activated microglia in A549 cell BM

**Fig. 3:**
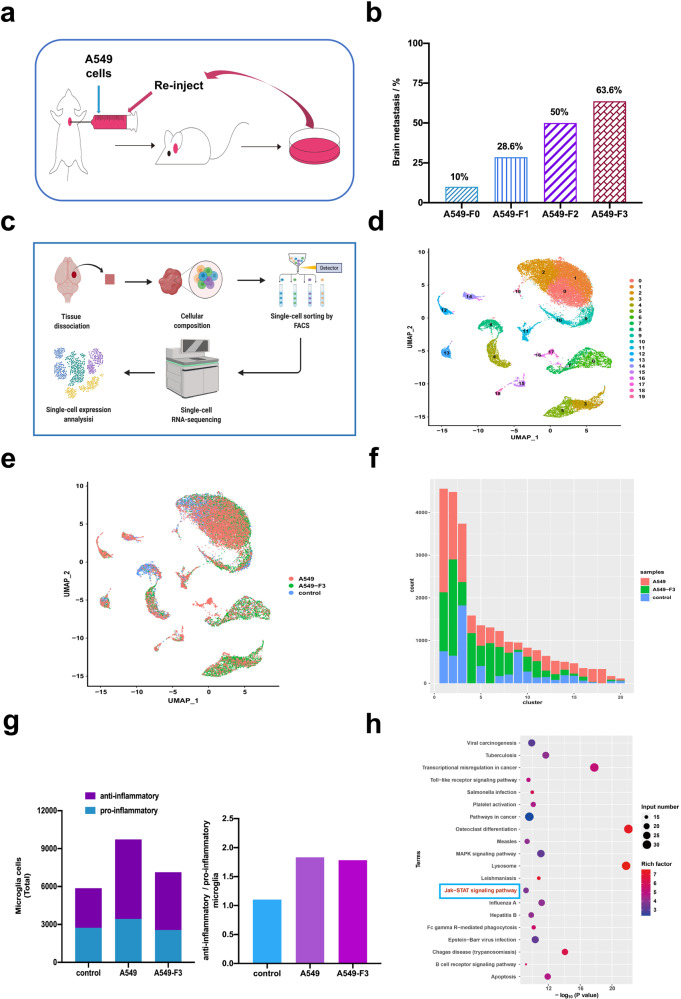
Single-Cell RNA-Seq Reveals activated microglia in metastatic lesions. **a** Schematic representation of the in vivo selection process. Parent A549 cells were inoculated into the left cardiac ventricle of nude mice. Tumor cells were isolated from brain lesions and reinoculated after expansion in culture. Cells isolated from the second round of metastases were expanded in culture and reinoculated to get the third-generation cells. **b** Histogram showed incidence of brain metastases in different cell lines (parental: 10.0%; A549-F1: 28.6%; A549-F2: 50.0%; A549-F3: 63.6%). **c** Schematic overview of the experimental design for the single-cell RNA-seq analyses was generated from the online tool (BioRender: https://biorender.com/). **d** UMAP analyzed cells reveal the existence of 20 clusters within the three experimental groups (untreated mouse brain, brain metastases from A549 and A549-F3 cells). **e** UMAP plots indicated distribution of cell clusters in different groups. **f** The numbers of different cell types in each group. **g** The numbers and percentages of anti-inflammatory and pro-inflammatory microglia in different groups. **h** The top 20 signal pathways with significant difference showed in KEGG

To further delineate the potential mechanism underlying the cellular communication between NSCLC BM cells and activated microglia, we firstly obtained highly brain-metastatic NSCLC cells by inoculating A549 cells into the left cardiac ventricles of male BALB/c nu/nu mice to isolate populations targeting the brain (Fig. 3a). We extracted cells from metastatic lesions and expanded them in culture for three rounds of in vivo selection (Supplementary Fig. S2a). The metastatic activity of luciferase-transduced A549 (Luc-A549) cells was confirmed by bioluminescence imaging (BLI), as well as by histological analysis of hematoxylin and eosin-stained brain-metastatic lesion sections (Supplementary Fig. S2b, c). The extracted cells, A549-F1, A549-F2 and A549-F3 were obtained in a first, second and third round of in vivo selection, respectively. Of note, the incidence of BM increased with the greater number of selections in vivo, with the A549-F3 cells having the highest final incidence of BM (63.6%; 7/11). In detail, the BM incidence increased from 10.0% at F0, to 28.6% at F1 and 50.0% at F2, respectively (Fig. 3b). A series of in vitro experiments were conducted to analyze malignant phenotypes of different cell lines. The colony formation test revealed no significant differences among these four derivative subpopulations (*P* > 0.05) (Supplementary Fig. S2d). Transwell invasion and wound-healing assays demonstrated that the invasiveness and migration activities of derivative cells were increased compared to parent A549-F0 cells (*P* < 0.05) (Supplementary Fig. S2e, f). Collectively, these observations confirmed that metastatic A549-F3 cells had an enhanced ability to invade and metastasize compared to the parent cells.

To characterize activated microglia involved in brain-metastatic colonization, we sorted microglia by single-cell RNA-seq and analyzed the difference of cell clusters in brains of the metastatic and healthy group using an index-sorting strategy, enabling the retrospective analysis of surface marker combinations for each individual cell (Fig. 3c). We identified 28165 cells in total with 11305 cells from A549-BM, 10459 cells from A549-F3-BM and 6401 cells from the tissue of the normal brain. The principal component analysis (PCA) in the three groups is shown in Supplementary Fig. S3a. Next, we characterized 20 cell clusters (cluster 0–19) among different cell types (Fig. 3d). The vast majority of cells were microglia (cluster 0, 1, 2, 3, 5, 9, 10, 16, 17, 19), and most cell clusters overlapped between metastatic lesions from A549 and A549-F3 cells (Supplementary Fig. S3b; Fig. 3e, f). We analyzed the top 10 differential genes in each cluster by random selection of 100 cells via Heat Map (Supplementary Fig. S3c). Then we further analyzed activated anti-inflammatory microglia in different groups based on the canonical cell markers listed in Supplementary Table S1. Results revealed that characteristic anti-inflammatory microglia were the dominant subgroup in the A549 and A549-F3 groups, while the anti-inflammatory and pro-inflammatory microglia showed similar numbers to maintain equilibrium state in the control group (Fig. 3g). The Kyoto Encyclopedia of Genes and Genomes (KEGG) pathway was applied to analyze the overlapped cluster of microglia in brain metastases. It’s showed that the JAK/STAT pathway and several other pathways were significantly upregulated (Fig. 3h). The upregulation of the JAK/STAT pathway is known to be involved in TAM activation. Cumulatively, based on these results, we confirmed the presence of M2 microglia in NSCLC-BM in vivo.

### IL6 emerged as a mediator of A549-F3 and M2 microglia communication in BM

**Fig. 4:**
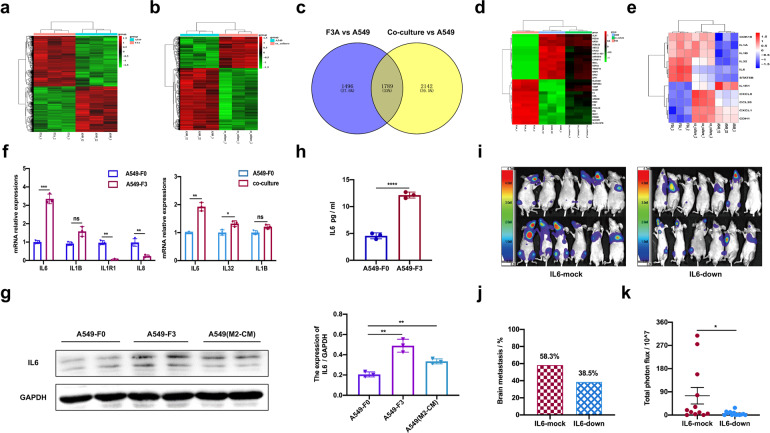
IL6 emerged as for a mediator of A549-F3 and M2 microglia communication in BM. **a** Heat map showed different gene expression profiles of A549-F3 populations and parental cells. **b** Heat map showed different gene expression profiles of A549 co-cultured with HMO6 for 48 h and parental cells. **c** Venn diagram of genes positively correlated with brain metastasis analyzed through transcriptome microarrays between parent A549 cells and A549-F3 or co-cultured A549 cells. **d** Comparison of mRNA microarray data showed top 30 changed genes (up or down) with the same expressed pattern between parent A549 cells and A549-F3 or co-cultured A549 cells. **e** Heat map of the most prominently upregulated cytokine in A549-F3 cells and co-cultured system. **f** qRT-PCR analysis showed expression of IL6 in different cell lines. **g** Western blot showed expression of IL6 in different cell lines. **h** ELISA assay of IL6 in culture supernatants from A549 and A549-F3 cells. **i** Brain metastases were determined by bioluminescence imaging. **j** Histogram showed incidence of brain metastases in different cell lines. **k** Photon flux detected from brain-metastatic focus in the different groups. Data are mean ± SD. *P < 0.05; **P < 0.01; ***P < 0.001

Comparative transcriptomic microarray analysis was performed to compare the genome-wide expression profiles of highly metastatic A549-F3 cells and A549-F0 cells as well as co-cultured with HMO6 microglia with that of parent A549 cells. The results revealed 4857 differentially expressed genes (DEGs) in BM-derived A549-F3 cells, and 1769 DEGs in co-cultured A549 cells (Fig. 4a, b). To further prioritize candidate genes, we conducted correlation analysis with two sets of gene chips based on standardized data (Supplementary Fig. S3d) and obtained a Venn diagram (Fig. 4c). The top 30 DEGs were for further analyzed (Fig. 4d). Overlapping genes with concordant expression trend were analyzed using KEGG pathway and gene ontology (GO) enrichment analysis (Supplementary Fig. S3e, f). IL6 was the most prominently upregulated cytokine in A549-F3 and co-cultured cells (Fig. 4e). The results of qRT-PCR and western blot confirmed elevated IL6 expression in A549-F3 and co-cultured A549 cells (Fig. 4f, g). Moreover, ELISA revealed that the level of IL6 was higher in A549-F3 cell media compared to that in A549 media (Fig. 4h). To confirm the effect of IL6 on BM, A549-F3/mock or IL6/knockdown cells were intracardially injected into nude mice (Supplementary Fig. S3g, h; Fig. 4i). After four weeks, metastatic activity was assayed using an in vivo BLI signal. Knockdown of IL6 led to a reduced incidence of BM, from 58.3% (7 in 12) to 38.5% (5 in 13) as well as weaker photon flux of metastatic lesions (Fig. 4j, k). These results suggested the importance of IL6 in A549-F3 BM and indicated the corresponding involvement of IL6-related signaling.

### IL6/JAK2/STAT3 signaling mediates M2 polarization of microglia

**Fig. 5:**
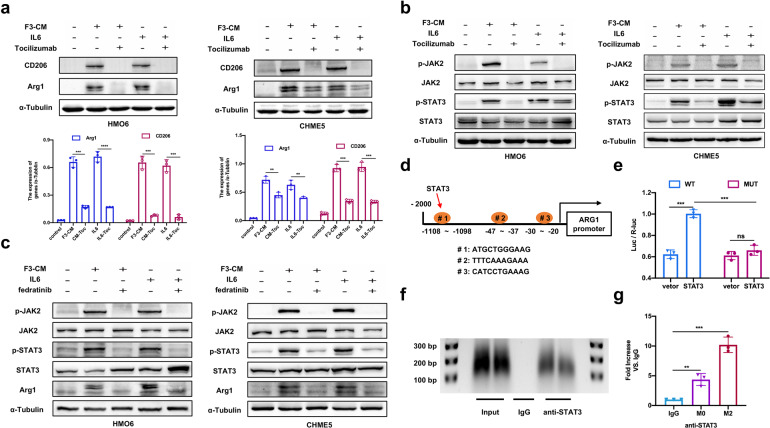
IL6/JAK2/STAT3 signaling mediates M2 polarization of microglia. **a** Western blot analysis of M2-marks (CD206 and Arg1) in HMO6 and CHME5 cells with control media or F3-CM alone or F3-CM treated with Tocilizumab (2.5 μg/ml for 24 h) or with IL6 (200 ng/ml for 24 h) alone or IL6-supplemented with Tocilizumab. **b** Western blot analysis of p-JAK2, JAK2, p-STAT3, and STAT3 in HMO6 and CHME5 cells with control media or F3-CM alone or F3-CM treated with Tocilizumab (2.5 μg/ml) or with IL6 (200 ng/ml) alone or IL6-supplemented with Tocilizumab. **c** Western blot analysis of CD206, Arg1, p-JAK2, JAK2, p-STAT3, and STAT3 in HMO6 and CHME5 cells with control media or F3-CM alone or F3-CM treated with fedratinib (20 μm for 24 h) or plus IL6(200 ng/ml) alone or IL6-supplemented with fedratinib. **d** The speculative binding sites in Arg1 for STAT3 were showed. **e** The dual-luciferase reporter experiment was applied to detect the luciferase activity of Arg1 stimulated by STAT3. **f** ChIP analysis was performed using a negative control immunoglobulin G (IgG) or anti-STAT3 antibody in CHME5 cells. **g** qPT-PCR of ChIP analysis was conducted to show binding site of STAT3 in ARG1. F3-CM: conditioned media of A549-F3 cells. Vector: pGL3-vector; STAT3: STAT3-vector. WT: wild type (#1); MUT: mutant type. M0:CHME5 cells; M2: F3-CM induced M2-CHME5 cells. Data are mean ± SD. *P < 0.05; **P < 0.01; ***P < 0.001

IL6 has been shown to activate the JAK/STAT signaling pathway, critically contributing to TAM polarization.^28,29^ Given that A549-F3 and co-cultured cells exhibited upregulated IL6, we speculated that IL6/JAK/STAT signaling might be involved in microglia M2 polarization. To dissect the detail of IL6 downstream signaling, we first sought to investigate the IL6-mediated M2 polarization at the protein level. Recombinant human IL6 at different concentrations was added into the culture medium of HMO6 and CHME5 cells (Supplementary Fig. S4a). Western blot analysis revealed that IL6 had the same effect as F3-CM, significantly upregulating M2 markers (CD206 and Arg1). Furthermore, tocilizumab, a monoclonal anti-IL6R neutralizing antibody, was used to study whether F3-CM induced M2 polarization through IL6 signaling. After adding tocilizumab at different concentrations to F3-CM or the IL6-supplemented culture media, the expression level of CD206 and Arg1 decreased in HMO6 and CHME5 cells (Fig. 5a; Supplementary Fig. S4b). Next, the expression levels of JAK/STAT pathway members such as JAK1, JAK2, STAT3, and STAT1, were analyzed. HMO6 or CHME5 cells could be stimulated via adding IL6 or F3-CM, whereas the levels of both phosphorylated JAK2 and STAT3 were reduced under the condition of tocilizumab treatment (Fig. 5b and Supplementary Fig. S4c). To investigate the role of JAK2/STAT3 signaling in M2 polarization, we treated HMO6 or CHME5 cells with the JAK2 inhibitor fedratinib. As expected, JAK2/STAT3 activation was suppressed via fedratinib in the presence of IL6 or F3-CM and the Arg1 expression level was correspondingly downregulated (Fig. 5c; Supplementary Fig. S4d, e). In summary, these results indicated that F3-CM-induced M2-polarization requires the activation of IL6/JAK2/STAT3 signaling.

Subsequently, we investigated whether ARG1 or CD206 expression could be directly regulated by STAT3. We used ECR Browser databases as well as TargetScan (http://www.targetscan.org/) to predict and identify ﻿that the promoter region of the *ARG1* gene containing conserved STAT3-binding sites showing in Fig. 5d. We selected the most conserved binding site (#1) for detailed analysis.

To confirm the functional link between the STAT3-binding site, dual-luciferase reporter vectors were constructed with a wild-type (#1) or mutant ARG1-promoter region and subsequently co-transfected with STAT3-vector or pGL3-vector into CHME5 cells. Overexpression of STAT3 resulted in increased expression of the ARG1-promoter-WT reporter gene, but it did not increase the expression of the ARG1-promoter-MT reporter gene (Fig. 5e). Next, the chromatin immunoprecipitation (ChIP)-PCR analysis was performed in CHME5 cells to identify the precise consensus sequences for ARG1-promoter activation. The putative STAT3-binding site (-1108 to -1098 bp) exhibited significant enrichment after immunoprecipitation with an anti-STAT3 antibody while no band was evident with the negative control IgG antibody (Fig. 5f). The qRT-PCR result indicated that the protein/ARG1 gene complexes were pulled down by the anti-STAT3 antibody in CHME5 cells (Fig. 5g). Our results indicated that *ARG1* might be a potential target gene of STAT3, mediating IL6-induced M2 polarization.

### Targeting IL6/JAK2/STAT3 signaling for suppression of A549-F3 metastasis in vivo

**Fig. 6:**
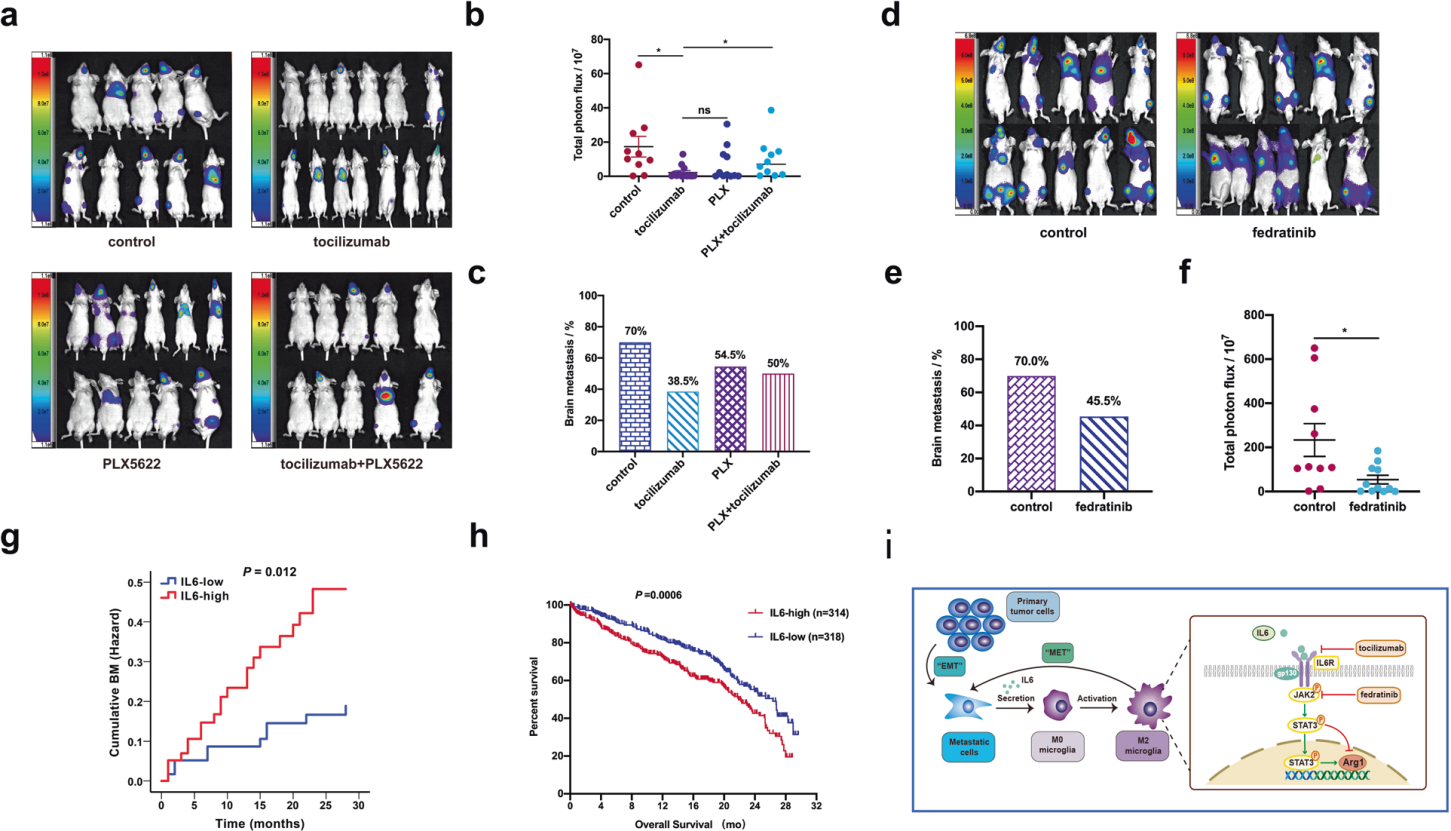
Targeting IL6/JAK2/STAT3 signaling for suppression of A549-F3 metastasis in vivo. **a** Brain metastases were determined by bioluminescence imaging. **b** Histogram showed incidence of brain metastasis in the different groups. **c** Photon flux detected from brain-metastatic focus in the different groups. **d** Brain metastases were determined by bioluminescence imaging. **e** Histogram showed incidence of brain metastasis in the different groups. **f** Photon flux detected from brain-metastatic focus in the different groups. **g** Kaplan–Meier estimates of the cumulative probability of brain metastasis among. NSCLC patients according to the level of IL6 in serum. **h** Kaplan–Meier survival curves of NSCLC patients with IL6 high expression and IL6 low expression. **i** Proposed model for IL6/JAK2/STAT3 activation in M2-microglia to promote colonization of brain-metastatic cells. Data are mean ± SD. *P < 0.05; **P < 0.01

Our in vitro data supported the key role of IL6 in M2 polarization, raising the question of whether IL6 inhibition could suppress the activity of JAK2/STAT3 signaling in microglia, which could subsequently reduce A549-F3 brain metastasis in vivo. We divided nude mice into four groups and injected them with A549-F3 cells in the left ventricle to establish BM. Thereafter, the four groups were treated with tocilizumab (5 mg/kg) by intraperitoneal injection once a week, treated daily with 12,000 mg/kg PLX5622, the combination of both inhibitors and phosphate-buffered saline (PBS) as a control (Supplementary Fig. S5a). Live imaging revealed a BM incidence of 38.5% (5 in 13) in the tocilizumab treatment group versus 70.0% (7 in 10) in the control group, while the BM incidence was higher in the combined treatment group (50.0%, 5 in 10) compared to the tocilizumab treatment group (Fig. 6a, b). Consistent with this result, the total BLI signal intensity of metastatic foci was the lowest in the group treated with tocilizumab alone (Fig. 6c). This indicates that the effect of tocilizumab on reducing BM incidence was significantly inhibited when microglia were depleted by PLX5622. Additional mice were randomly divided into two groups, and BM were established as previously described. The treatment group received fedratinib (100 mg/kg) and the control group received corn oil by gavage once every two days (Supplementary Fig. S5b). At the end time point, BM burden in the treatment group was significantly lower than that in the control group (45.5% [5 in 11] versus 70% [7 in 10]), along with lower luminescence from the metastatic foci (*P* < 0.05; Fig. 6d-f). Tocilizumab or fedratinib-treated mice experienced less metastasis-related morbidity and markedly lower body weight loss compared to those in the control group (Supplementary Fig. S5c). Meanwhile, the serum IL6 was higher in brain-metastatic groups than mice without BM (Supplementary Fig. S5d). Furthermore, ﻿tissues from the brain-metastatic lesion were used for immunohistochemistry (IHC) analysis. Results revealed that p-STAT3 and Arg1 were mainly expressed at the brain stroma, with weak expression in metastatic lesions. Microglia were labeled with IBA1 and Tmem119 (Supplementary Fig. S6a). Taken together, these results demonstrated that IL6 inhibitors (tocilizumab or fedratinib) can block JAK2/STAT3 activation and impede BM of A549-F3 cells in vivo.

### Higher IL6 in serum is associated with higher risk of BM and poor prognosis

To further confirm the clinical value of IL6 in NSCLC-BM, we collected peripheral blood samples from 120 NSCLC patients without BM (Supplementary Table S2) and evaluated the feasibility of predicting BM by IL6 level in serum. These patients were divided into two groups by IL6 level (*n* = 120). After 28 months of follow-up, the two groups were analyzed for differences in BM. We found the two groups had different rates of BMs and the group with higher IL6 levels had a higher risk of brain metastasis (35% vs 17%, *P* = 0.012) (Fig. 6g). Although no survival data was available due to limited follow-up time, TCGA data revealed that NSCLC patients with a low IL6 level (*n* = 318, median survival time = 21.87 months) had longer overall survival than those with high IL6 (*n* = 314, median survival time = 26.33 months; *P* = 0.0006) (Fig. 6h). Further experiments have shown that the expression of IL6 and p-STAT3 in brain-metastatic tissue from NSCLC patients is much higher than patients without metastasis (Supplementary Fig. S6b). Overall, these results indicated that IL6 promotes NSCLC-BM in vivo and may act as a predictive biomarker of BM in NSCLC patients.

Based on the above results and previous studies, we wanted to further analyze the relationship between *JAK2* gene polymorphism and the risk of brain metastasis in NSCLC patients. Kaplan–Meier survival analysis of *JAK2* rs10815144 showed that GA/AA genotypes had a higher cumulative probability of brain metastasis (23% vs. 7%, *P* = 0.014) (Supplementary Fig. S6c). Collectively, we summarized our study in a schematic diagram (Fig. 6i).

## Discussion

Brain metastases are associated with dismal prognosis for limited effective treatment options. Due to the unique microenvironment in the brain, the development of NSCLC-BM is a highly intricate and selective process.^30^ Thus, it’s urgent to reveal molecular mechanisms of NSCLC-BM and provide novel therapy strategies to improve patient’s prognosis. Our study showed that brain-specific metastatic A549-F3 cells induced M2 microglia via the IL6/JAK2/STAT3 signaling pathway and the skewed M2 microglia promoted NSCLC-BM development by influencing the colonization of metastatic cells. Further results indicated IL6 level in serum is associated with the occurrence and development of brain metastases in NSCLC patients. Our use of tocilizumab or fedratinib exhibiting activity to inhibit M2-microglia polarization in vitro and reduce the occurrence of brain metastases of A549-F3 cells in vivo. The current research is the first to show the prophylactic use of tocilizumab has the potential to reduce the incidence of BM in high-risk NSCLC patients.

Given the heterogeneity of the primary tumor, only a minority of cells have the ability to successfully form BM. In order to study this population of metastatic cells and based on the methods of isolation of cells with tissue-metastatic tropism, we established a cell line (A549-F3) selectively metastatic to the brain.^31,32^ We found that A549-F3 cells exhibited a mesenchymal phenotype and this finding confirmed the importance of cancer cells with mesenchymal phenotypes in the development of metastasis.^33^ But the mesenchymal phenotypes is insufficient and may even impede early colonization for that metastatic tumor cells need to restore some of the epithelial characteristics required for metastatic formation. The mesenchymal-to-epithelial transition (MET) in metastatic foci is a complex process induced by the metastatic microenvironment including cell-intrinsic or stromal components.^11^ As essential components of the brain’s immune microenvironment, microglia possess macrophage characteristics and can affect the development of brain metastases. However, direct mechanistic evidence that clarify interaction between microglia and cancer cells during NSCLC-BM is still lacking. Here, we found that microglia were dominated by M2 phenotype in brain metastases of mice or NSCLC patients. In vitro, we discovered that A549-F3 cells could polarize microglia cells into M2-phenotype, in turn, M2-microglia activated by metastatic cells facilitate MET of mesenchymal cancer cells, which is essential for metastatic colonization in the brain. Interestingly, Watabe et al. recently showed the critical impact of nicotine on lung cancer-BM by skewing microglia to M2-phenotype and this study is consistent with our findings on the role of M2-microglia.^34^

The functional plasticity of macrophages/microglia has been a point of controversy, but the current consensus is that phenotype determines function.^35,36^ Our single-cell RNA-sequencing analysis revealed that M2-microglia was the dominant cell population in the microenvironment within NSCLC-BM. Classical M1-like and alternative M2-like phenotypes of macrophages/microglia are determined by the presence of different cytokines, including interferon-γ, GM-CSF, IL-4, and M-CSF.^37–39^ The IL6 was recognized as the most highly upregulated cytokine in brain-metastatic A549-F3 cells and induced activation of M2-microglia in the paracrine pattern. Previous studies have reported the role of IL6 in macrophage polarization,^40,41^ we conducted a series of in vitro experiments. Exogenous addition of recombinant IL6 or co-culture conditioned medium was sufficient to activate M2-microglia, while added with inhibitors (tocilizumab and fedratinib) to target JAK2/STAT3 signaling showing the ability to impede the formation of M2-microglia. These results directly demonstrated that IL6 secreted by metastatic A549-F3 cells promoted the M2-microglia polarization via the JAK2/STAT3 pathway.

As an anti-IL6R antibody, tocilizumab has been FDA-approved for the treatment of rheumatic disease and has recently been evaluated for the treatment of solid tumors in clinical trials.^42–44^ Our study reconfirmed that targeting IL6R could reduce NSCLC-BM.^45^ Considering the direct role of tocilizumab on lung cancer cells, we explored the effect of tocilizumab in inhibiting brain metastases following microglial depletion with PLX5622. Results showed that the incidence of brain metastases in the tocilizumab treatment group and the combined treatment group (tocilizumab and PLX5622) were 38.5% and 50%, respectively. Thus, we verified that tocilizumab could reduce brain metastases attributing to inhibition of IL6 signaling in activated microglia. Interestingly, a recent study indicated ﻿that unspecific inhibition of CX3CR1 targeting macrophages/microglia might not be a suitable therapeutic option to prevent brain metastases of lung cancer. Herein, our findings suggested that patients may benefit from targeting of specifically activated microglia.^46^ In addition, our application of fedratinib successfully inhibited JAK2/STAT3 signaling and reduced the BM of A549-F3 cells in nude mice. This is critically important since numerous JAK2 inhibitors are currently in active clinical development for hematopoietic proliferative disorders and malignancies.^47,48^ Future work should examine the potential of JAK2 inhibitors as adjuvant therapy for reducing BM in NSCLC patients.

As we all know, increased serum IL6 levels uniformly appear to correlate with advanced stages and poor survival independent of the cancer type.^49,50^ However, there is scarcely evidence that IL6 level is associated with the occurrence and development of brain metastases in NSCLC patients. We analyzed incidence of BM in NSCLC patients with different serum IL6 levels, and results showed the group with higher IL6 levels had a higher risk of brain metastasis (35% vs. 17%, *P* = 0.012). Our study indicated that serum IL6 levels have the potential to predict brain metastases in NSCLC patients, which might be an important component of predictive model for NSCLC-BM. At present, there is a lack of intervention methods to prevent brain metastasis in NSCLC. This research provides a new predictor for screening high-risk of brain metastasis in NSCLC patients and new preventive interventions for NSCLC-BM to improve patient’s survival. The current findings present new perspectives for the development of treatment regimens in cancers basing on targeting innate immunity. To our knowledge, this is the first study to demonstrate that inhibitors (tocilizumab and fedratinib) can interfere with the occurrence of NSCLC-BM by targeting microglia. The study provides theoretical evidence for the clinical application of these inhibitors in future phase II clinical studies.

Nevertheless, the current study has some limitations. First, the mechanism of IL6/JAK2/STAT3 signaling in M2 macrophage polarization warrants further elucidation. Second, the efficacy of the investigated inhibitors must to be further validated in the application of clinical trials.

In conclusion, we have provided a detailed molecular analysis of the reciprocal communication between brain-metastatic cells (A549-F3) and activated M2-microglia. Moreover, our results highlight IL6 as a prognostic factor for the occurrence of BM and a potential therapeutic target of prophylactic treatment in NSCLC-BM. Novel potential prevention and treatment approaches have been explored to modulate the microglial phenotype through the IL6/JAK2/STAT3 signaling pathway. With the rapid advent of immunotherapy in cancer treatment, we believe NSCLC-BM patients will benefit from the treatment with tocilizumab or fedratinib.

## Materials and methods

### Patient samples

All patients (*n* = 282) in our study had histologically confirmed NSCLC treated at our hospital between 2016 and 2020. There were no restrictions on age, sex, or disease stage, but peripheral blood samples had to be available for analysis. Clinical information was obtained from the patients’ medical records. These patients were divided into two groups, in one group (*n* = 120, Supplementary Table S2) patients firstly diagnosed with NSCLC excepted for patients with BM or immune-related diseases were used for ELISA of IL6 level in serum. Peripheral venous blood samples were collected when patients were diagnosed with non-small lung cancer but had not received any treatment. Then the samples were centrifuged at 2000 rpm for 20 min at 4 °C and the supernatant frozen at −80 °C. As for IL6 levels in patients, the cut-off value of 7.85 pg/ml was determined by median concentration in all samples. Accordingly, the patients (*n* = 120) were divided into two groups with high and low IL6 level. The other group (*n* = 162, Supplementary Table S3) was genotyped with peripheral blood lymphocytes for single nucleotide polymorphisms (SNPs) of genes. The specific analysis of SNPs referred to our previous studies.^51^ Five pairs of paraffin-embedded primary lung cancer tissues and paired brain-metastatic tissues were used for immunohistochemistry analysis. The IL6 gene expression of NSCLC patients from the Cancer Genome Atlas (TCGA) database are available from the Cancer Genomics Browser of University of California Santa Cruz (https://genome-cancer.ucsc.edu/). Written informed consent was obtained from patients before sample collection, and all related procedures were approved by the Ethics Committee of Tongji Medical College (IRBID: TJ-C20121219 and TJ-IRB20210222).

### Animal studies

All animal experiments were conducted following the National Institutes of Health guide for the care and use of Laboratory animals and approved by the Ethics Committee of the Institutional Animal Care and Use Committee of Tongji Medical College, Huazhong University of Science and Technology. Male BALB/c nude mice (6–8-week-old, Institute of Laboratory Animal Science) were housed under specific pathogen-free conditions. For brain metastasis assays, 100 μl PBS containing 5 × 10^5^ luciferase-labeled A549 (Luc-A549) cells was inoculated into the left cardiac ventricle of mice using 26 G needles. Mice were anesthetized with sodium pentobarbital (1.5%, 50 mg/kg) before manipulation. Brain metastasis was confirmed by in vivo bioluminescence imaging. Briefly, mice received an intraperitoneal injection of 150 μl of 1.5% D-luciferin and anaesthetized with 1.5% sodium pentobarbital. Imaging was completed with a Xenogen IVIS system (Spectral Instrument Imaging, Lago X) coupled to Living Image Acquisition and Analysis software. We chose 4 weeks after the injection as the endpoint. To evaluate the effect of inhibitors on the incidence of BM, mice were randomly divided into four groups. Tocilizumab (5 mg/kg in 100 μl PBS) was administered by intraperitoneal injection one day before the intracardiac injection of A549-F3 cells. Thereafter, tocilizumab was administered intraperitoneally once a week for 4 weeks, while control mice received 100 μl PBS. For depletion of microglia, mice were administered dietary PLX5622 (the inhibitor of colony-stimulating factor-1 receptor, 1200 mg/kg feed; M18090601, Moldiets, biopike.inc, China) from one week before the start of the experiment to the end of the experiment. Fedratinib (JAK2 inhibitor; 100 mg/kg in 10% DMSO and 90% corn oil) was administered to experimental group mice through oral gavage once every two days for 4 weeks. Control group mice received corn oil. The incidence of brain metastasis was quantified and analyzed by intravital imaging based on bioluminescent signals in the brain. Peripheral blood was collected from the orbital venous plexus of mice at the endpoint to analyze IL6 level in serum by ELISA.

### Cell culture and reagents

NSCLC cell lines (A549, H292, H1299, and H460) were obtained from the Oncology Laboratory of Tongji Hospital (Wuhan, China) and cultured in RPMI-1640 (Hyclone, Provo, UT) supplemented with 10% fetal bovine serum (FBS; Gibco, MD, USA). Human microglia cell lines CHME-5 and HMO6 (purchased from the Wuhan University School of Medicine, Wuhan, China) were grown in Dulbecco’s modified Eagle’s medium (DMEM, HyClone) with 10% FBS. All cells were cultured in an incubator at 37 °C in humidified air with 5% CO_2_. For bioluminescent tracking, A549 cells were stably transfected with firefly luciferase with an antibiotic resistance gene (Genechem, Shanghai, China). Puromycin was purchased from GeneChem. Recombinant human TGF-β1 (100-21 C) and IL6 were purchased from PeproTech (Rocky Hill, NJ, USA). Fedratinib (JAK2 inhibitor) was purchased from MedChemExpress (Monmouth Junction, NJ). Tocilizumab, an anti-human neutralizing IL6R antibody, was obtained from Selleckchem (Tokyo, Japan). All products were stored and used according to manufacturer instructions.

### Co-culture experiments and collection of conditioned media

For co-culture experiments, non-contact cells were co-cultured in 6-well plates with a 0.4 μm pore polyester membrane insert (Corning; New York, USA). Contact cells were co-cultured in 25-cm^2^ culture flasks. A549 cells were co-cultured with HMO6 or CHME5 cells at a ratio of 1:5. To obtain conditioned media, A549, HMO6, CHME5 or co-cultured cells were cultured for 48 h. Then cells conditioned medium (ACM, HCM, CCM, co-HCM, and co-CCM) was collected, centrifuged for 15 min at 2000 rpm, and filtered (0.45 μm, Whatman GmbH, Dassel, Germany).

### Isolation and purification of brain-metastatic cells

Using bioluminescence imaging, we selected mice with BM. Lesions were resected under sterile conditions after sacrifice. Brain metastasis tissues were minced and placed in a 1:1 mixture of RPMI-1640 and 0.2% collagenase I. The samples were incubated at 37 °C in a humidified incubator for about 1 h and gently blown once every 20 min. Cells were then briefly centrifuged at a low speed (1200 rpm for 5 min), resuspended in 2 ml 0.25% trypsin, and incubated for a further 10 min. The cells were collected, centrifuged, and resuspended in RPMI-1640 supplemented with 10% FBS and 1% penicillin-streptomycin. Cells were allowed to grow and adhere to 25-cm^2^ culture flasks. Brain-metastatic cells were selected with puromycin, and purified metastatic cells (A549-F1) were propagated in culture before re-introduction into mice to obtain A549-F2 and A549-F3 cells.

### Microarray data analysis

A549-F0 and A549 cells were co-cultured with HMO6 cells for 48 h, and A549-F3 cells were collected for total RNA extraction using TRIzol Reagent (Takara, Shiga, Japan). mRNA expression profiling was performed using the Affymetrix GeneChip Human 1.0 ST arrays according to the manufacturer’s protocol, and raw expression data were generated at the microarray core facility of the National Cancer Institute (Frederick, MD). The raw data were filtered based on *P* < 0.05 and a fold change greater than 1.2 or less than 0.8333. Functional analysis was conducted via Gene Ontology (GO) and KEGG (Kyoto Encyclopedia of Genes and Genomes) enrichment.

### Quantitative real-time PCR (qRT-PCR)

Total RNA from cells was extracted using Trizol Reagent (Takara, Shiga, Japan). After detection of RNA concentration (NanoDrop 2000c UV Spectrophotometer; Thermo Fisher Scientific, Wilmington, DE), 1 μg of total RNA was reverse transcribed into cDNA using PrimeScript RT Reagent kit (RR037A; Takara). The cDNA was used for subsequent qRT-PCR using the SYBR Premix Ex Taq (RR420A; Takara) and running on the 7900HT Fast real-time PCR system (Thermo Fisher). For data analysis, the cycle time (Ct) values of the selected genes were first normalized with the value of β-actin of the same samples, and then the relative expression was calculated using the 2^-ΔΔCt^ method. Three replicate PCR amplifications were performed for each sample. The sequences of primers are enlisted in the Supplementary Table S4.

### Western blot

Western blot was performed as previously published. Cells were lysed using radioimmunoprecipitation assay (RIPA; Beyotime, Shanghai, China) buffer. The total protein concentration was measured using a BCA Protein Assay Kit (AR0197; Boster, Wuhan, China). Equal amounts of total protein were separated by SDS-PAGE and transferred to 0.25 μm (for IL6) or 0.45 μm (polyvinylidene fluoride membranes) PVDF membranes (Millipore, Billerica, USA). Primary antibodies included anti-α-tubulin (1:8000, 60004-1-Ig; Proteintech); anti-GAPDH (1:10,000, 60004-1-Ig; Proteintech); anti-CD206 (1:1000, 9139, Cell Signaling Technology); anti-iNOS (1:1000, ab32101; Abcam), anti-Arg1 (1:10,00, 93668; Cell Signaling Technology), anti-IL6R (1:1000, 60004-1-Ig; Proteintech), and anti-IL6 (1:1000, 9139, Cell Signaling Technology). After washing thrice with TBST, the membranes were incubated with an HRP-conjugated (horseradish peroxidase-conjugated; the Promoter Biotechnology, Wuhan, China) secondary antibody at a 1:10000 dilution for 1 h at room temperature. Antibody binding was detected using SuperSignal West Pico Chemiluminescent Substrate (Thermo Scientific). Experiments were repeated at least 3 times, and band intensities were quantified using ImageJ software.

### Dual-luciferase reporter assay

In brief, the full-length human ARG1 cDNA was ligated into the promoter-driven luciferase reporter plasmid pGL4.10. The luciferase reporter plasmids containing wild (pGL4.10-promoter ARG1-WT) or mutant (pGL4.10-promoter ARG1-MUT) type ARG1-promoter sequences were constructed by Heyuan Co. (Shanghai, China). The *Renilla* luciferase vector plasmids (pcDNA3.1) and pEnter-STAT3 (pcDNA3.1-STAT3) vector were co-transfected into the HMO6 cells using Lipofectamine 2000 (11668019, Invitrogen, USA). At 48 h after transfection, we performed luciferase assays using a luciferase reporter assay system (E1960, Promega, USA). Luciferase/*Renilla* luciferase activity was used to calculate luciferase activity using a fluorescence microscope (MHG-100B, MOTIC, USA).

### Chromatin immunoprecipitation (ChIP)

For the ChIP experiment, we used the SimpleChIP Enzymatic Chromatin IP Kit (9003, Cell Signaling Technology, USA) according to the manufacturer’s instructions. Briefly, treated CHME5 cells were added to 1% formaldehyde and incubated for protein-DNA complexes. After terminating the reaction, cells were washed twice and collected with ice-cold PBS containing a protease inhibitor for lysing. Lysates were centrifugated at 2000 rpm for 5 min, resuspended, and sonicated to obtain 150 to 900 bp DNA fragments. The protein-DNA lysates were added with antibodies against normal rabbit IgG, STAT3 (Cell Signaling Technology, USA), and incubated with rotation overnight at 4 °C. Next, protein A agarose beads were added into the lysates and mixed for 2 h. After washing, the complexes were reverse cross linked at 65 °C for 2 h in the presence of 0.2 M NaCl and Proteinase K. Subsequently, the precipitated DNA was purified using a centrifugal column. A total of 2 μl of the purified DNA was subjected to PCR amplification using primers that were derived from the Arg1 promoter: (forward) 5’-AAGCTCGACGGTTAAGTGGA-3’ and (reverse) 5’-TGAAGTCTCATCATTGGTGCCA-3’). The amplified DNA included the predicted STAT3-binding site of -1108 to -1098. Meantime, the PCR products were analyzed by electrophoresis on a 1% agarose gel to confirm the DNA fragments within the range of 150–900 bp. The results were analyzed by expression as enrichment relative to input.

### Enzyme-Linked Immunosorbent Assay (ELISA) analysis

The levels of IL6 secreted by the cells in the medium and in serum of NSCLC patients were determined with a QuantiCyto® Human IL6 ELISA kit (EHC007; NeoBioscience, China) according to the manufacturer’s instruction. In brief, CM was harvested after 24 h of culture in serum-free media and serum from blood samples of patients was separated by low-speed centrifugation. The optical densities were measured at 450 nm with an enzyme-labeling instrument (ELX800, BioTek, USA) and calculated as pg/mL.

### Single-cell RNA-seq

To obtain microglia, brain tissue was dissociated using a gentle MACS Dissociator (Miltenyi Biotec, Germany) and digested with 0.5 mg/mL collagenase type IV (Promoter Biotechnology, China)) and 0.02 mg/mL DNase I (D8071; Solarbio, China) in RPM-1640 medium at 37 °C for 45 min with gentle shaking. The digested brain suspension was filtered and homogenized with a 70 μm diameter cell strainer (HBLK-151040; Sorfa, China). The cell suspension was separated by 30%/70% layered Percoll (17-0891-09; Cytiva, USA) gradient centrifugation at 400 × *g* for 30 min at 25 °C with low acceleration and no brake. The intermediate layer was slowly collected and washed with PBS. Sequencing libraries were generated using the 10X Genomics Chromium Controller and analyzed using Cell Ranger (Version 3.1.0). Major clusters were denoted by differentially expressed canonical marker genes via UMAP (Uniform Manifold Approximation and Projection) and subjected to additional rounds of cluster refinement. Differentially expressed genes (|log FoldChange | > 0.25) were screened with the *bimod* statistical test.

### Genomic DNA extraction and polymorphism genotyping

Genomic DNA was isolated from peripheral blood lymphocytes by using a Quick Gene DNA Whole Blood Kit S (Thermo Scientific, USA) according to the manufacturer’s protocol, and stored at −80 °C. The SNPs were genotyped by using matrix-assisted laser desorption/ionization-time of flight (MALDI-TOF) mass spectrophotometry to detect allele-specific primer extension products with the MassARRAY platform (Sequenom, Inc., USA). Assay data were analyzed using Sequenom TYPER software (version 4.0).

### Lentivirus transduction, cell immunofluorescence, tissue immunohistochemistry and immunofluorescence staining, colony formation assay, Transwell invasion assay, and wound-healing migration assay

Detailed information is provided in the Supplementary Materials and Methods.

### Statistical analysis

All analyses, unless specified otherwise, were performed using GraphPad Prism8.0.1 (GraphPad Software, CA, USA). Data are presented as the mean ± standard deviation (SD). For comparison between two groups, unpaired student’s *t* test was performed, whereas one-way analysis of variance (ANOVA) followed by post hoc Dunnett’s multiple comparison test was used to compare significant differences among more than two groups. The Kaplan–Meier survival analysis was performed using the log-rank test. A Cox proportional hazards model was applied to calculate hazard ratios (HRs) and 95% confidence intervals (CIs) to evaluate the influence of genotypes on the risk of brain metastasis performed with the SPSS software (version 23.0). Significant differences were represented as *P < 0.05, **P < 0.01, ***P < 0.001, ****p < 0.0001 unless otherwise indicated.

## Supplementary information

Supplementary Materials

## Data Availability

All the data generated or analyzed during this study are included in this published article and its supplementary files.

## References

[1] Sung H (2021). Global cancer statistics 2020: GLOBOCAN estimates of incidence and mortality worldwide for 36 cancers in 185 countries. CA Cancer J Clin..

[2] Kastner J, Hossain R, White CS (2020). Epidemiology of Lung Cancer. Semin Roentgenol..

[3] Wood SL, Pernemalm M, Crosbie PA, Whetton AD (2014). The role of the tumor-microenvironment in lung cancer-metastasis and its relationship to potential therapeutic targets. Cancer Treat. Rev..

[4] Khalifa J (2016). Brain Metastases from NSCLC: Radiation Therapy in the Era of Targeted Therapies. J. Thorac. Oncol..

[5] Arrieta O (2009). Brain metastasis development and poor survival associated with carcinoembryonic antigen (CEA) level in advanced non-small cell lung cancer: a prospective analysis. BMC Cancer..

[6] Coppes-Zantinga AR, Coppes MJ (2000). Sir James Paget (1814-1889): a great academic Victorian. J. Am. Coll. Surg..

[7] Yin W (2020). BBB-penetrating codelivery liposomes treat brain metastasis of non-small cell lung cancer with EGFR(T790M) mutation. Theranostics.

[8] Wang H (2017). TGF-beta1-induced epithelial-mesenchymal transition in lung cancer cells involves upregulation of miR-9 and downregulation of its target, E-cadherin. Cell Mol. Biol. Lett..

[9] Lu W, Kang Y (2019). Epithelial-Mesenchymal Plasticity in Cancer Progression and Metastasis. Dev. Cell..

[10] Yao D, Dai C, Peng S (2011). Mechanism of the mesenchymal-epithelial transition and its relationship with metastatic tumor formation. Mol. Cancer Res..

[11] Jolly MK (2017). EMT and MET: necessary or permissive for metastasis?. Mol. Oncol..

[12] Bigagli E, Cinci L, D’Ambrosio M, Luceri C (2019). Transcriptomic Characterization, Chemosensitivity and Regulatory Effects of Exosomes in Spontaneous EMT/MET Transitions of Breast Cancer Cells. Cancer Genomics Proteom..

[13] Gunasinghe NPAD, Wells A, Thompson EW, Hugo HJ (2012). Mesenchymal–epithelial transition (MET) as a mechanism for metastatic colonisation in breast cancer. Cancer Metastasis Rev..

[14] Shibue T, Weinberg RA (2011). Metastatic colonization: settlement, adaptation and propagation of tumor cells in a foreign tissue environment. Semin Cancer Biol..

[15] Izraely S (2019). The metastatic microenvironment: Melanoma-microglia cross-talk promotes the malignant phenotype of melanoma cells. Int J. Cancer.

[16] Brandenburg S (2015). Resident microglia rather than peripheral macrophages promote vascularization in brain tumors and are source of alternative pro-angiogenic factors. Acta Neuropathologica..

[17] Gutmann DH, Kettenmann H (2019). Microglia/Brain Macrophages as Central Drivers of Brain Tumor Pathobiology. Neuron.

[18] Hambardzumyan D, Gutmann DH, Kettenmann H (2016). The role of microglia and macrophages in glioma maintenance and progression. Nat. Neurosci..

[19] Reitman ZJ (2019). Mitogenic and progenitor gene programmes in single pilocytic astrocytoma cells. Nat. Commun..

[20] He BP (2006). Differential reactions of microglia to brain metastasis of lung cancer. Mol. Med..

[21] Tardito S (2019). Macrophage M1/M2 polarization and rheumatoid arthritis: A systematic review. Autoimmun. Rev..

[22] Locati M, Curtale G, Mantovani A (2020). Diversity, Mechanisms, and Significance of Macrophage Plasticity. Annu. Rev. Pathol.: Mechanisms Dis..

[23] Solinas G, Germano G, Mantovani A, Allavena P (2009). Tumor-associated macrophages (TAM) as major players of the cancer-related inflammation. J. Leukoc. Biol..

[24] Allavena P (2008). The inflammatory micro-environment in tumor progression: the role of tumor-associated macrophages. Crit. Rev. Oncol. Hematol..

[25] Pukrop T (2010). Microglia promote colonization of brain tissue by breast cancer cells in a Wnt-dependent way. Glia.

[26] Wu Y, Dissing-Olesen L, MacVicar BA, Stevens B (2015). Microglia: Dynamic Mediators of Synapse Development and Plasticity. Trends Immunol..

[27] Chen XF (2012). Transforming growth factor-beta1 induces epithelial-to-mesenchymal transition in human lung cancer cells via PI3K/Akt and MEK/Erk1/2 signaling pathways. Mol. Biol. Rep..

[28] Cho H (2018). Cancer-Stimulated CAFs Enhance Monocyte Differentiation and Protumoral TAM Activation via IL6 and GM-CSF Secretion. Clin. Cancer Res..

[29] Fu XL (2017). Interleukin 6 induces M2 macrophage differentiation by STAT3 activation that correlates with gastric cancer progression. Cancer Immunol. Immunother..

[30] Achrol AS (2019). Brain metastases. Nat. Rev. Dis. Prim..

[31] Bos PD (2009). Genes that mediate breast cancer metastasis to the brain. Nature.

[32] Nguyen DX (2009). WNT/TCF signaling through LEF1 and HOXB9 mediates lung adenocarcinoma metastasis. Cell.

[33] Pastushenko I, Blanpain C (2019). EMT Transition States during Tumor Progression and Metastasis. Trends Cell Biol..

[34] Wu, S. Y. et al. Nicotine promotes brain metastasis by polarizing microglia and suppressing innate immune function. *J Exp Med*. **217**, e20191131 (2020). 10.1084/jem.20191131 PMC7398164 32496556

[35] Eggen BJL, Raj D, Hanisch UK, Boddeke HWGM (2013). Microglial Phenotype and Adaptation. J. Neuroimmune Pharmacol..

[36] Ransohoff RM (2016). A polarizing question: do M1 and M2 microglia exist?. Nat. Neurosci..

[37] Ma R (2016). Tumor cell-derived microparticles polarize M2 tumor-associated macrophages for tumor progression. Oncoimmunology.

[38] Mia S (2014). An optimized protocol for human M2 macrophages using M-CSF and IL-4/IL-10/TGF-beta yields a dominant immunosuppressive phenotype. Scand. J. Immunol..

[39] Draijer C, Penke LRK, Peters-Golden M (2019). Distinctive Effects of GM-CSF and M-CSF on Proliferation and Polarization of Two Major Pulmonary Macrophage Populations. J. Immunol..

[40] Yin Z (2018). IL-6/STAT3 pathway intermediates M1/M2 macrophage polarization during the development of hepatocellular carcinoma. J. Cell Biochem..

[41] Sanmarco LM (2017). IL-6 promotes M2 macrophage polarization by modulating purinergic signaling and regulates the lethal release of nitric oxide during Trypanosoma cruzi infection. Biochim Biophys. Acta Mol. Basis Dis..

[42] Scott LJ (2017). Tocilizumab: A Review in Rheumatoid Arthritis. Drugs.

[43] Kampan NC (2018). Immunotherapeutic Interleukin-6 or Interleukin-6 Receptor Blockade in Cancer: Challenges and Opportunities. Curr. Med Chem..

[44] Wadstrom H, Frisell T, Askling J, Anti-Rheumatic Therapy in Sweden Study G (2017). Malignant Neoplasms in Patients With Rheumatoid Arthritis Treated With Tumor Necrosis Factor Inhibitors, Tocilizumab, Abatacept, or Rituximab in Clinical Practice: A Nationwide Cohort Study From Sweden. JAMA Intern Med..

[45] Noda M (2012). IL-6 receptor is a possible target against growth of metastasized lung tumor cells in the brain. Int J. Mol. Sci..

[46] Zhang W (2021). In vivo two-photon characterization of tumor-associated macrophages and microglia (TAM/M) and CX3CR1 during different steps of brain metastasis formation from lung cancer. Neoplasia.

[47] Mullally A (2010). Physiological Jak2V617F expression causes a lethal myeloproliferative neoplasm with differential effects on hematopoietic stem and progenitor cells. Cancer Cell..

[48] Roskoski R (2020). Properties of FDA-approved small molecule protein kinase inhibitors: A 2020 update. Pharm. Res..

[49] Chang Q, Daly L, Bromberg J (2014). The IL-6 feed-forward loop: a driver of tumorigenesis. Semin Immunol..

[50] Lippitz BE, Harris RA (2016). Cytokine patterns in cancer patients: A review of the correlation between interleukin 6 and prognosis. Oncoimmunology.

[51] Li QX (2017). The Thr300Ala variant of ATG16L1 is associated with decreased risk of brain metastasis in patients with non-small cell lung cancer. Autophagy.

