# Supplementary material for: Targeting polarized phenotype of microglia via IL6/JAK2/STAT3 signaling to reduce NSCLC brain metastasis
Source: Signal Transduct Target Ther. 2022 Feb 23;7:52. doi: 10.1038/s41392-022-00872-9 (PMC8864012; doi:10.1038/s41392-022-00872-9)
Supplement: Supplementary file 1 — Supplementary Materials [file 41392_2022_872_MOESM1_ESM.docx]

**Supplementary Materials for**

**Targeting polarized phenotype of microglia via IL6/JAK2/STAT3 signaling to reduce NSCLC brain metastasis**

Yu Jin1^1#^, Yalin Kang^1#^, Minghuan Wang^2#^, Bili Wu^1^, Beibei Su^1^, Han Yin^1^, Yang Tang^1^, Qianxia Li^1^, Wenjie Wei^1^, Qi Mei^1^, Guangyuan Hu^1^, Lukacs-Kornek Veronika^3^, Jian Li^3^, Kongming Wu^1^, Xianglin Yuan^1*^ and Wei Wang^2*^

^1^ Department of Oncology, Tongji Hospital, Tongji Medical College, Huazhong University of Science and Technology, Wuhan 430030, Hubei Province, China

^2^ Department of Neurology, Tongji Hospital, Tongji Medical College, Huazhong University of Science and Technology, Wuhan 430030, Hubei Province, China

^3^ Institute of Experimental Immunology, University Clinic of Rheinische Friedrich-Wilhelms-University, Bonn, Germany

**These authors contributed equally**: Yu Jin, Yalin Kang, Minghuan Wang

**Correspondence to**: Xianglin Yuan, e-mail: yuanxianglin@hust.edu.cn and Wei Wang, e-mail: wwang@vip.126.com.

**This file includes:**

Materials and Methods

Supplementary Text

Supplementary Figures. S1 to S6

Supplementary Tables S1 to S4

**Supplementary Materials and Methods**

***Lentivirus transduction***

Cells were seeded in a 12-well plate and cultured for 12–24 h to 50-60% confluence in complete medium before transfection. The culture medium was replaced with serum-free medium containing lentivirus (Heyuan, Shanghai, China) and polybrene (5 μg/mL). As a negative control, cells were transduced with an empty vector. After 8–12 h incubation, serum-free medium was changed for complete medium. Transfection efficiency was observed under a fluorescence microscope 48–72 h after infection. Cells were treated with 5 µg/mL puromycin for 7–10 d to select stably transfected cells. IL6 downregulation was confirmed by real-time qRT-PCR and western blot.

***Cell immunofluorescence***

The expression of E-cadherin and vimentin in A549 cells and CD206 in microglia was analyzed by immunofluorescence. Briefly, cells were plated on glass slides in 24-well culture plates at a concentration of 3×10^4^ cells/well and treated as required. The cells were then washed with PBS and fixed in 4% formaldehyde solution for 10 min, followed by permeabilization with 0.05% Triton X-100 (ST795, Beyotime Biotechnology, China) in PBS, followed by three washes with PBS. Next, the cells were blocked with 10% BSA at 37 °C for 1 h and then stained with primary antibodies against E-cadherin (1:100, 20874-1-AP; Proteintech), vimentin (1:150; ab8978; Abcam, UK), and CD206 (1:50, 18704-1-AP; Proteintech, China) at 4 °C overnight. Secondary antibodies conjugated to Alexa Fluor 488 and FITC (catalog A-21203 and A-21208; 1:150; Promoter Biotechnology, China) were used to visualize primary antibody binding. Finally, cells were counterstained with DAPI for 5 min, and fluorescent images were visualized under a confocal laser scanning microscope (DMI3000B; Leica Microsystems, China)

***Tissue immunohistochemistry﻿ and immunofluorescence staining***

Hematoxylin and eosin (H&E), immunohistochemistry, and immunofluorescence staining were conducted following standard procedures. ﻿Primary antibodies used for immunohistochemistry included: anti-IBA1 (1:8000, ab178847; Abcam, UK) and anti-IL6 (1:1600, 66146-1-Ig; Proteintech, Wuhan). Primary antibodies for immunofluorescence included: anti-IBA1 (1:8000, ab178847; Abcam, UK) and anti-Arg1 (1:800, 93668; Cell Signaling Technology, USA). ﻿Tissues were counterstained with DAPI. Images of each stained slide were randomly taken using an inverted microscope (DMI3000B, Leica, Germany). At least three randomly selected fields were used for statistical analysis.

***Colony formation assay***

For the colony formation assay, different descendant cells in the logarithmic growth phase were plated in 6-well plates (2000 cells/well) and cultured for 2 weeks. Colonies were fixed in 4% paraformaldehyde for 20 min and stained with 0.1% crystal violet for 15 min. After washing and air-drying, the clones were counted under an inverted microscope (cells > 50) in at least 3 different fields. The colony formation rate was calculated, and every treatment group included three duplicate wells for statistical analysis. Colony formation rate was calculated using the following formula: colony formation rate = (number of colonies formed / number of seeded cells) ×100%.

***Transwell invasion assay***

We used 24-well Transwell plates with 8μm pore filters (Corning, Corning, USA) for invasion assays. The inserts were pre-coated with 50 μl of diluted Matrigel (RPMI-1640, 1:9). The filters were air-dried and hydrated with 60 μl serum-free RPMI-1640. Approximately 5×10^4^ cells were suspended in 200 μl of RPMI-1640 medium with 1% FBS and placed in the upper chamber, while 600 μl RPMI-1640 medium with 20% FBS was added to the lower chamber. After 48 h of incubation, cells were fixed in 4% paraformaldehyde for 30 min and stained with 0.1% crystal violet for 20 min. Next, non-invading cells on the upper surface were removed. Cells on the underside of filters were counted and photographed (×200 magnification) in five microscopic fields. All experiments were performed at least three times.

***Wound-healing migration assay***

Cells were seeded in 12-well plates and grown to a confluent monolayer for about 12-24 h. The monolayer was scratched with a 20 μl pipette tip. The cells were then washed with PBS and cultured in serum-free RPMI1640 medium for 48 h. Migration was photographed and recorded at 0 and 48 h under a microscope. We measured the migration distances using ImageJ analysis software (National Institutes of Health, Bethesda, MD). The scratch healing rate was calculated as: (0 h width of scratch - 48 h width of scratch)/0 h width of scratch × 100%. Each experiment was performed in triplicate.

**Supplementary Figures**

**Figure S1**

**
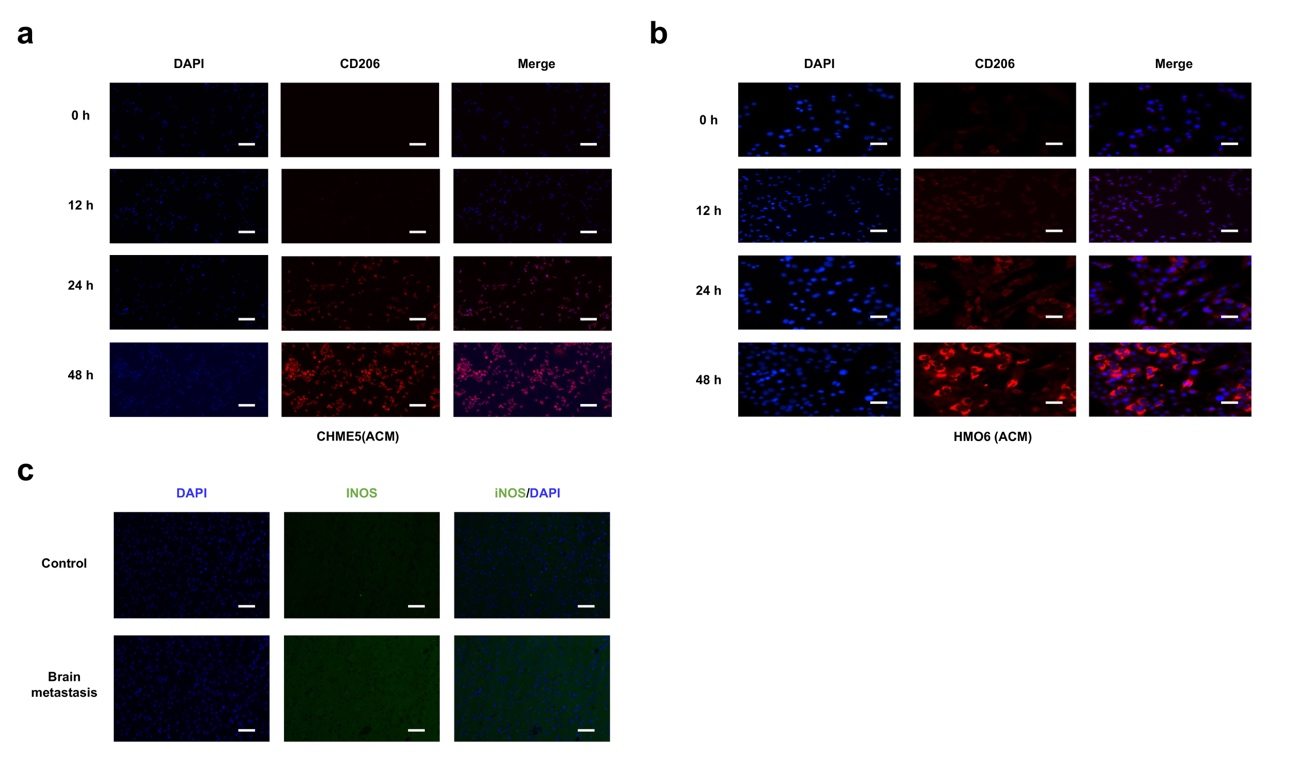
The immunofluorescent staining of microglia.**

(**a**) Immunofluorescent staining of CD206 indicated M2 polarization in CHME5 cells after treated with ACM for 0, 12, 24 and 48 h. (magnification: 100×). (**b**) Immunofluorescent staining of CD206 indicated M2 polarization in HMO6 cells after treated with ACM for 0, 12, 24 and 48 h. (magnification: 200×). (**c**) Representative images of immunofluorescence staining with DAPI (blue) for nuclei and iNOS (green) for M1-mark were depicted (magnification: 20×). The red signal represents the staining of CD206, the green signal represents the staining of iNOS and the blue signal represents the DAPI-stained nuclei.

**Figure S2**

**
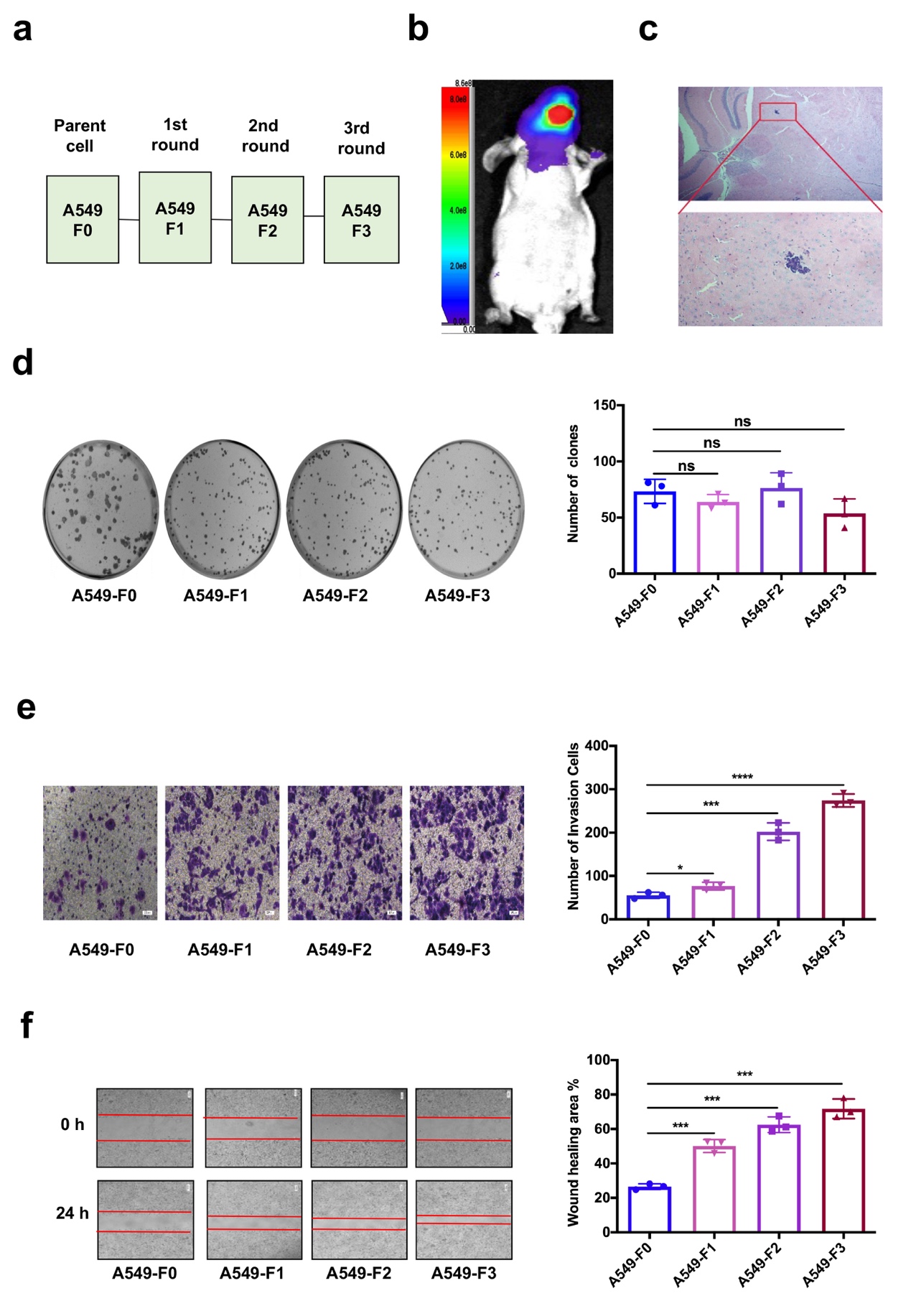
In vivo selection of brain metastatic derivatives from the parent A549 cells.**

(**a**) Flowcharts of the *in-vivo*-selected brain metastatic derivatives and describing the nomenclature for cell lines. (**b**) Brain metastatic lesions were detected by intravital imaging analysis after 4-weeks inoculation of mice with luciferase-labeled parental A549 cells (stably transfected with luciferase-labeled gene). (**c**) Representative images of brain metastases and H&E of brain tissues were presented. The scale bar represents 20 μm. (**d**) The cell proliferation capacity of different cell lines was analyzed by colonies analysis. (**e**) Evaluating the ability of invasion in different cell lines by Transwell invasion assay (magnification, 400×). (**f**) Images and quantification of wound healing assay. (magnification, 40×). Data are mean ± SD. *P*>0.05 no significant difference; **P*<0.05; ***P*<0.01; ****P*<0.001; *****P*<0.0001.

**Figure S3**


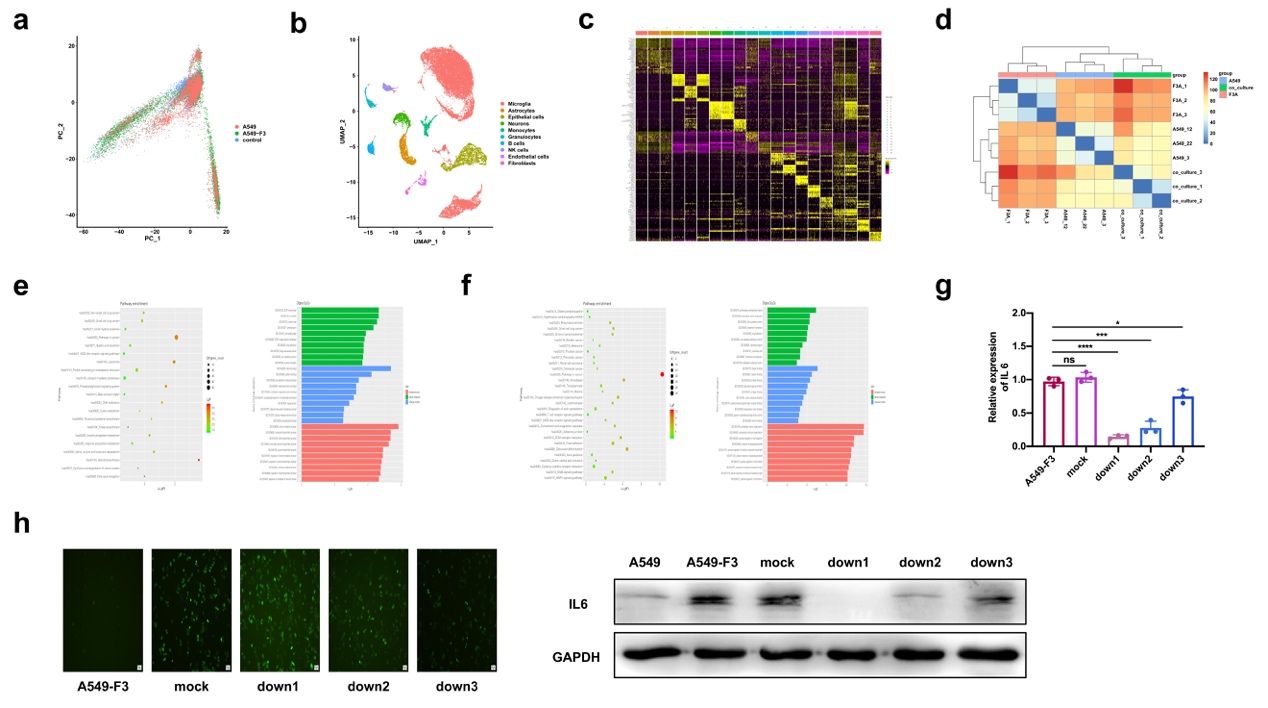
**The analysis of single-sell** **RNA-Seq and** **transcriptome RNA-Seq.**

(**a**) Using PCA (Principal Component Analysis) to reduce dimensionality of data and show distribution of the main component in different groups. (**b**) UMAP plots indicated distribution of cell types in different groups. (**c**) TOP10 genes of 100 cells were randomly selected for gene expression in each cluster showing in heat map. (**d**) Correlation analysis of different samples based on standardized data. (**e**) Overlapping genes with concordant expression trend were analyzed using KEGG pathway. (**f**) Overlapping genes with concordant expression trend were analyzed using gene ontology (GO) enrichment analysis. (**g**) qRT-PCR analysis showed expression of IL6 in different cell lines. (**h**) Western blot confirmed that IL6 was downregulated in IL6/knockdown cell lines.Data are mean ± SD. *P*>0.05 no significant difference; **P*<0.05; ***P*<0.01; ****P*<0.001; **** *P*<0.0001.

**Figure S4**

**
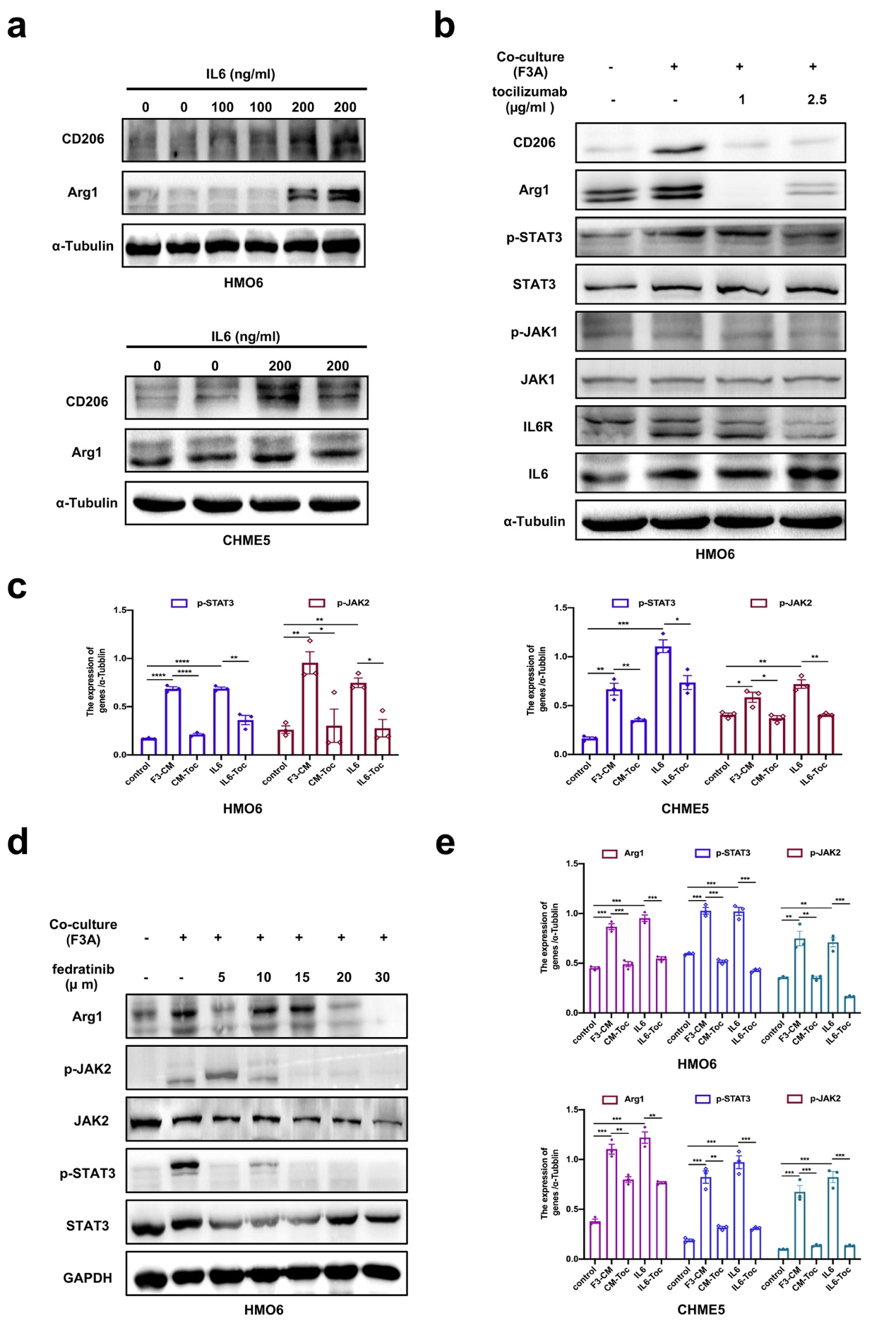
The Western blot analysis of HMO6 and CHME5 cells.**

(**a**) HMO6 and CHME5 cells were treated with different concentrations (0, 100 and 200 ng/ml) of IL6 for 24 h, and the expression of Arg1 and CD206 was evaluated by Western blot. (**b**) HMO6 cells were treated with different concentrations (0, 1 and 2.5 μg/ml) of tocilizumab for 24 h, and the expression of Arg1, CD206, JAK2, p-JAK2, STAT3, p-STAT3, JAK1, p-JAK1, STAT1, p-STAT1 and IL6 R was evaluated by Western blot. (**c**) The gray value analysis of Western blot. (**d**) HMO6 cells were treated with different concentrations (0, 5, 10, 15, 20 and 30 μm/ml) of fedratinib for 24 h, and the expression of Arg1, JAK2, p-JAK2, STAT3 and p-STAT3 was evaluated by Western blot. (**e**) The gray value analysis of Western blot.

**Figure S5**


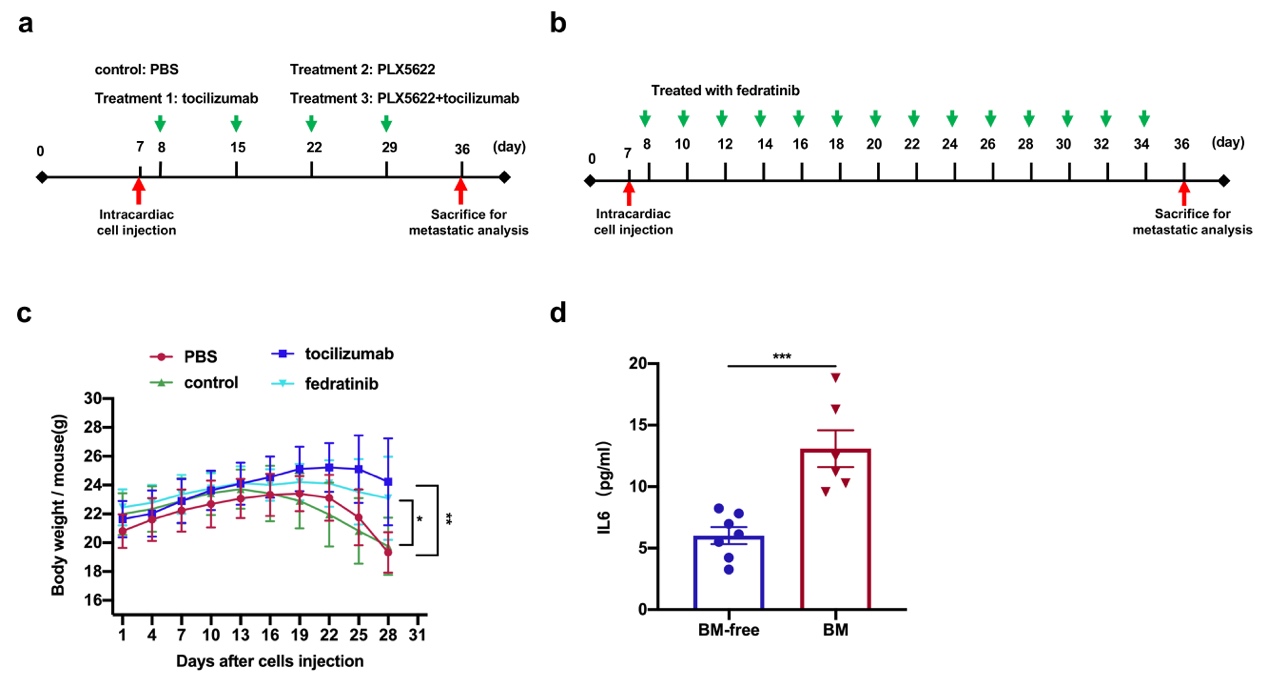
**The analysis of animal experiments.**

(**a**) Schematic diagram illustrates the treatment schedule of Tocilizumab and PLX5622. (**b**) Schematic diagram illustrates the treatment schedule of fedratinib. (**c**) Average body weight of mice in different groups over time following cells inoculation (PBS, n =10; tocilizumab, n =13; control, n= 10; fedratinib, n =11). (**d**) ELISA analysis showed the IL6 in serum of mice with or without brain metastases. Data are mean ± SD. *P*>0.05 no significant difference; **P*<0.05; ***P*<0.01; ****P*<0.001.

**Figure S6 ﻿**


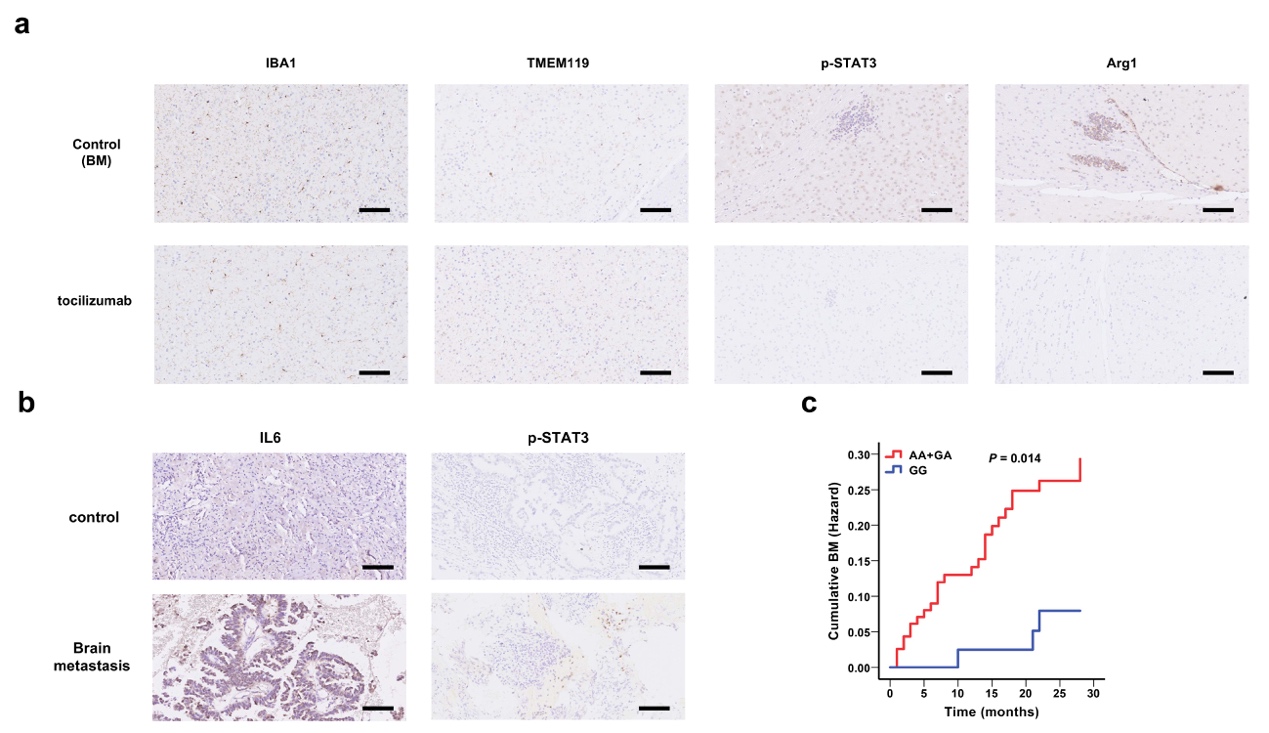
**The images of IHC staining and the Kaplan-Meier analysis of the SNP genotypes**.

(**a**) ﻿Representative images showing IHC staining of IBA1, Tmem119, p-STAT3 and Arg1 from brain tissue of mouse. (**b**) Representative images showing IHC staining of IL6 and CD206 from brain tissue of lung cancer patients. (**c**) Kaplan-Meier estimates of the cumulative probability of brain metastasis among NSCLC patients according to the genotype of *JAK2* rs10815144. control (BM): brain metastatic tissue of control group; tocilizumab: group treated with IL6R inhibitor. control: normal brain tissue; brain metastasis: metastatic brain tissue. Scale bar, 200×.

**Supplementary Table S1** Markers of pro-inflammatory and anti-inflammatory microglia.

| **Gene ID** | **Official Symbol** |
| --- | --- |
| pro-inflammatory |  |
| ENSMUSG00000020826 | Nos2 |
| ENSMUSG00000022901 | Cd86 |
| ENSMUSG00000021242 | Npc2 |
| ENSMUSG00000002985 | Apoe |
| ENSMUSG00000040987 | Mill2 |
| ENSMUSG00000036905 | C1qb |
| ENSMUSG00000024397 | Aif1 |
| ENSMUSG00000027776 | Il12a |
| ENSMUSG0000000429 | Il12b |
| anti-inflammatory |  |
| ENSMUSG00000026712 | Mrc1 |
| ENSMUSG00000019987 | Arg1 |
| ENSMUSG00000008845 | Cd163 |
| ENSMUSG00000016529 | Il10 |
| ENSMUSG00000030579 | Tyrobp |
| ENSMUSG00000058715 | Fcer1g |
| ENSMUSG00000001131 | Timp1 |
| ENSMUSG00000023992 | Trem2 |
| ENSMUSG00000020027 | Socs2 |
| ENSMUSG00000020372 | Rack1 |
| ENSMUSG00000030844 | Rgs10 |
| ENSMUSG00000027447 | Cst3 |
| ENSMUSG00000062380 | Tubb3 |
| ENSMUSG00000022650 | Retnlb |
| ENSMUSG00000079293 | Clec7a |
| ENSMUSG00000031779 | Ccl22 |

**Supplementary Table S2** Summary of clinic-pathologic characteristics of patients.

| Characteristics | No. of patients | No. of events |
| --- | --- | --- |
| Total | 120 | 31 |
| Age |  |  |
| <60y | 78 | 23 |
| ≥60y | 42 | 8 |
| Sex |  |  |
| Male | 67 | 15 |
| Female | 53 | 16 |
| Histology |  |  |
| Squamous cell carcinoma | 32 | 6 |
| Adenocarcinoma | 85 | 24 |
| NSCLC-NOS | 3 | 1 |
| Clinical Stage |  |  |
| I-III | 64 | 16 |
| IV | 51 | 15 |
| Unable to evaluate | 5 | 0 |
| KPS |  |  |
| ≥ 80 | 119 | 31 |
| < 80 | 1 | 0 |

**Supplementary Table S3** Summary of clinic-pathologic characteristics of patients.

| Characteristics | No. of patients | No. of events |
| --- | --- | --- |
| Total | 162 | 30 |
| Age |  |  |
| <60y | 94 | 23 |
| ≥60y | 68 | 7 |
| Sex |  |  |
| Male | 103 | 16 |
| Female | 59 | 14 |
| Histology |  |  |
| Squamous cell carcinoma | 37 | 4 |
| Adenocarcinoma | 121 | 26 |
| NSCLC-NOS | 4 | 0 |
| Clinical Stage |  |  |
| I-III | 95 | 11 |
| IV | 62 | 17 |
| Unable to evaluate | 5 | 2 |
| KPS |  |  |
| ≥ 80 | 151 | 28 |
| < 80 | 11 | 2 |

**Supplementary Table S4** The primer sequences used for quantitative reverse transcription-polymerase chain reaction

| **Gene** | **Forward or Reverse** | **Sequences (5’-3’)** |
| --- | --- | --- |
| ACTB | Forward | CTGGAACGGTGAAGGTGACA |
|  | Reverse | AAGGGACTTCCTGTAACAATGCA |
| E-cadherin | Forward | AGGCCAAGCAGCAGTACATT |
|  | Reverse | ATTCACATCCAGCACATCCA |
| Vimentin | Forward | ACACCCTGCAATCTTTCAGACA |
|  | Reverse | GATTCCACTTTGCGTTCAAGGT |
| CD206 | Forward | GGGTTGCTATCACTCTCTATGC |
|  | Reverse | TTTCTTGTCTGTTGCCGTAGTT |
| Arg1 | Forward | GGAATCTGCATGGGCAACCTGTGT |
|  | Reverse | AGGGTCTACGTCTCGCAAGCCA |
| iNOS | Forward | TTCAGTATCACAACCTCAGCAAG |
|  | Reverse | TGGACCTGCAAGTTAAAATCCC |
| CD86 | Forward | GCTCGTAGTATTTTGGCAGGACC |
|  | Reverse | CGGGTATCCTTGCTTAGATGAGC |
| IL6 | Forward | TGCGTCCGTAGTTTCCTTCT |
|  | Reverse | GCCTCAGACATCTCCAGTCC |
| IL1B | Forward | AAGCTGAGGAAGATGCTG |
|  | Reverse | ATCTACACTCTCCAGCTG |
| IL1R1 | Forward | ATGAAATTGATGTTCGTCCCTGT |
|  | Reverse | ACCACGCAATAGTAATGTCCTG |
| IL8 | Forward | GAATGGGTTTGCTAGAATGTGATA |
|  | Reverse | CAGACTAGGGTTGCCAGATTTAAC |
| IL32 | Forward | TGGCGGCTTATTATGAGGAGC |
|  | Reverse | CTCGGCACCGTAATCCATCTC |
